# Preoperative measurement of the hiatal surface with MDCT: impact on surgical planning

**DOI:** 10.1007/s11547-021-01413-0

**Published:** 2021-08-27

**Authors:** Marco Rengo, Cristian E. Boru, Stefano Badia, Angelo Iossa, Davide Bellini, Simona Picchia, Nicola Panvini, Iacopo Carbone, Gianfranco Silecchia, Andrea Laghi

**Affiliations:** 1 Department of Medico-Surgical Sciences and Biotechnologies, Diagnostic Imaging Unit, Sapienza” University of Rome, ICOT Hospital, Via Franco Faggiana, 1668. 04100 Latina, Italy; 2 Department of Medico-Surgical Sciences and Biotechnologies, Division of General Surgery and Bariatric Centre of Excellence IFSO-EC, Sapienza” University of Rome, ICOT Hospital, Via Franco Faggiana, 1668. 04100 Latina, Italy; 3 Department of Radiological, Oncological and Pathological Sciences, Diagnostic Imaging Unit, “Sapienza” University of Rome, ICOT Hospital, Via Franco Faggiana, 1668. 04100 Latina, Italy; 4 Department of Surgical and Medical Sciences and Translational Medicine, Diagnostic Imaging Unit, “Sapienza” University of Rome, Sant’Andrea University Hospital, Via di Grottarossa, 1035. 00189 Rome, Italy

**Keywords:** Laparoscopic bariatric surgery, Gastroesophageal reflux, Multidetector computed tomography, Esophageal hiatus, Diaphragm, Hiatal hernia

## Abstract

**Objective:**

To evaluate the accuracy and reproducibility of hiatal surface area (HSA) measurement on dedicated multidetector computed tomography (MDCT) acquisition, in patients, previously subjected to laparoscopic sleeve gastrectomy (LSG), and affected by gastroesophageal reflux disease (GERD). Intraoperative HSA measurement was considered the reference standard.

**Methods:**

Fifty-two candidates for laparoscopic hiatal hernia repair were prospectively included in the study. MDCT images were acquired during swallowing of oral iodinated contrast media and during strain. Measurements were performed by nine readers divided into three groups according to their experience. Results were compared with intraoperative measurements by means of Spearman correlation coefficient. Reproducibility was evaluated with intra- and interreader agreement by means of weighted Cohen’s kappa and intraclass correlation coefficient (ICC).

**Results:**

Significant differences between MDCT and intraoperative HSA measurements were observed for swallowing imaging for less experienced readers (*p* = 0.037, 0.025, 0.028 and 0.019). No other statistically significant differences were observed (*p* > 0.05). The correlation between HSA measured intraoperatively and on MDCT was higher for strain imaging compared to swallowing (r = 0.94—0.92 vs 0.94—0.89). The overall reproducibility of MDCT HSA measurement was excellent (ICC of 0.95; 95% CI 0,8993 to 0,9840) independently of reader’s experience

**Conclusion:**

HSA can be accurately measured on MDCT images. This method is reproducible and minimally influenced by reader experience. The preoperative measurement of HSA has potential advantages for surgeons in terms of correct approach to hiatal defects in obese patient.

## Introduction

Laparoscopic sleeve gastrectomy (LSG) is an effective surgical treatment of morbid obese patients, providing a considerable and durable weight loss, as well as a resolution/improvement of related comorbidities [1]. However, LSG has been reported to increase the risk of “de novo” or recurrent gastroesophageal reflux disease (GERD), due to anatomical and pathophysiological changes [2].

An incidence of 6–13% of intrathoracic migration of the gastric sleeve (ITM) has been reported after a mean time of 12 months, ranging from 1 day to 3 years [3, 4]. ITM, similar to sliding hiatal hernia (HH), is characterized by a widening of the muscular hiatus and circumferential laxity of the phreno-esophageal ligament, allowing the esophagogastric junction and the upper part of the sleeve to herniate into the mediastinum [5, 6]. ITM is associated with GERD and increased incidence of severe esophagitis and Barrett esophagus [2–4, 7].

The surgical management of HH continues to pose a challenge to the surgeon [8], since widening of the hiatus has clinical implications, both for the choice of surgical repair’s method and for the long-term postoperative success rate [9]. The complex reciprocal relationship between the HH and hiatal defect has been investigated [10]. Whereas hiatal defect contributes to the HH’s pathogenesis, the herniated sleeve per se enlarges the hiatus, both causing impairment of the low esophageal sphincter (LES) function and predisposing to reflux. The concept of hiatal surface area (HSA) has been proposed to define the size of the hiatal defect, which should allow to determine the two-dimensional expanse of the hiatal orifice and then adapt the crural closure to the exact dimension of the hiatal orifice [11]. Thus, HSA measurement has been advocated as useful tool for choosing the right tailored treatment (simple or reinforced posterior cruroplasty) [11, 12].

Preoperative barium swallow examination’s sensitivity is very poor when compared with MDCT [8, 13–15]. A negative correlation between radiologic appearance of the sleeve’s migration and the development of GERD symptoms was found: The accuracy of standard barium radiographic studies is negatively influenced by its plain bidimensional nature especially if compared to MDCT [16]. MDCT demonstrated to be more accurate than the conventional radiology and endoscopy for the detection of morphological alteration causing GERD symptoms after LSG and was considered a valid noninvasive method to guide surgery and monitoring operated patients [16]. Moreover, HSA has been demonstrated to be measurable also with MDCT on a population of patients not priory subjected to surgery, affected by HH [13].

Thus, the primary aim of this study was to determine whether HSA can be measured on MDCT, also in patients previously subjected to sleeve gastrectomy and to evaluate its accuracy compared to surgical measurement. The secondary aim of the study was to evaluate the reproducibility of the imaging technique and if it is influenced by operator experience. The tertiary aim of the study was to investigate the correlation between HSA and other morphologic features evaluated on MDCT.

## Material and methods

### Study design and population

**Fig. 1:**
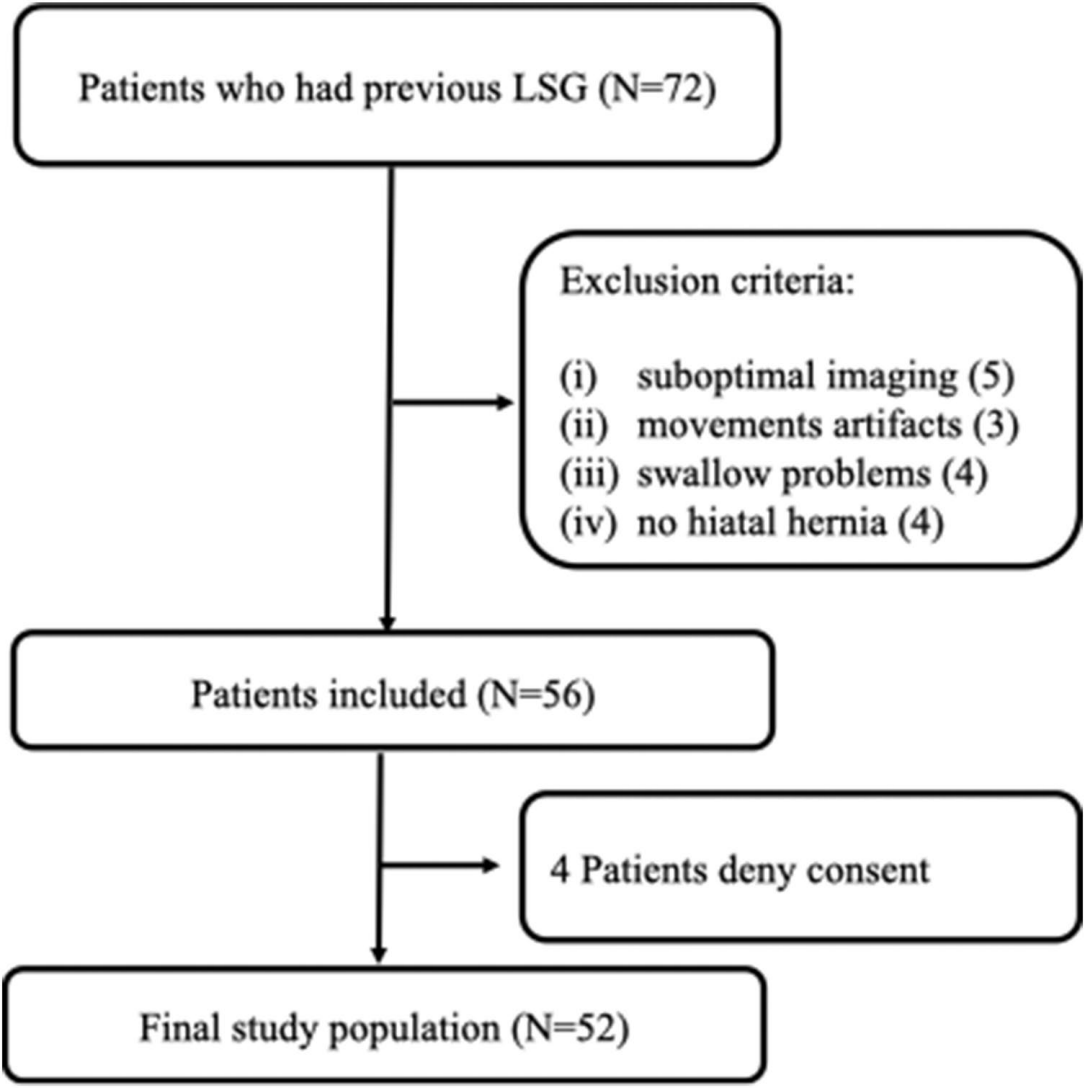
Flowchart of patient’s enrollment. LSG (laparoscopic sleeve gastrectomy)

This single-center prospective study started in March 2013 and ended in June 2020. Patients previously subjected to LSG, between 2011 and 2019, presenting postoperative GERD symptoms and/or HH, and candidates to revisional surgery were included. Patients’ recruitment was based on the Standards for Reporting of Diagnostic Accuracy (STARD) initiative as reported in accrual flowchart (Fig. 1). Indication for revisional surgery was persistent or recurrent GERD symptoms, despite treatment with proton pump inhibitors (PPI), ITM and/or insufficient weight loss (IWL). Upper gastrointestinal symptoms were assessed by two self-rating questionnaires: GERD Impact Scale [17] and GERD-Health Related Quality of Life (GERD-HRQL) [18]. Esophagogastroduodenoscopy (EGD) with multiple biopsies and Helicobacter pylori test was performed in all patients; esophagitis presence was classified according to the Los Angeles (LA) Classification [19]. During EGD, the presence of gastritis or other lesions was registered. pH metry and esophageal manometry completed the workup, in selected cases.

This Health Insurance Portability and Accountability Act-compliant study was approved by the Institutional Review Board (IRB), and informed consent was obtained. No authors are employees of or consultants for industry or had control of inclusion of any data and information that could represent a conflict of interest. There was no industry support specifically for this study.

### MDCT acquisition protocol

A dedicated acquisition protocol was performed in all patients to reproduce the physiological conditions of the gastroesophageal tract. Acquisitions were performed immediately after the oral administration of 500 ml of a 4% solution of iodinated contrast medium (sodium diatrizoate and meglumine diatrizoate solution, 370 mg/ml, Gastrografin®, Bayer Schering Pharma AG, Germany) and tap water. The first acquisition (swallowing) in craniocaudal direction, ranging from the skull base to the transverse umbilical line, was acquired during the ingestion of the last gulp of contrast medium. This acquisition was performed to obtain the distension of the esophagus as during swallowing. A second acquisition (straining) in caudocranial direction, ranging from the transverse umbilical line to the skull base, was acquired immediately after the end of the first acquisition during Valsalva maneuver. This acquisition was performed to evaluate the mobility of gastroesophageal junction (GEJ) and to reproduce a high intraabdominal pressure as during laparoscopy. Both acquisitions were performed during one single apnea. No spasmolytic agents were administered before the scan. All images were acquired with patients on supine position.

All exams were performed using a 64-row MDCT scanner (Lightspeed VCT®, GE Medical Systems, Waukesha, Wis, USA). Scanning parameters were adjusted as follows: kVp, 120; beam pitch, 1.375:1; detector configuration, 64 × 0.625 mm; reconstructed section thickness, 0.625 mm; standard reconstruction algorithm. A z-axis tube current modulation was used, with a noise index of 28 (min/max mA: 200/600) which was recommended by the manufacturer for standard abdominal CT. A 40% radiation dose reduction protocol was applied in all patients using iterative reconstructions (ASiR®, GE Medical Systems, Waukesha, WI, USA).

### Image analysis

**Fig. 2:**
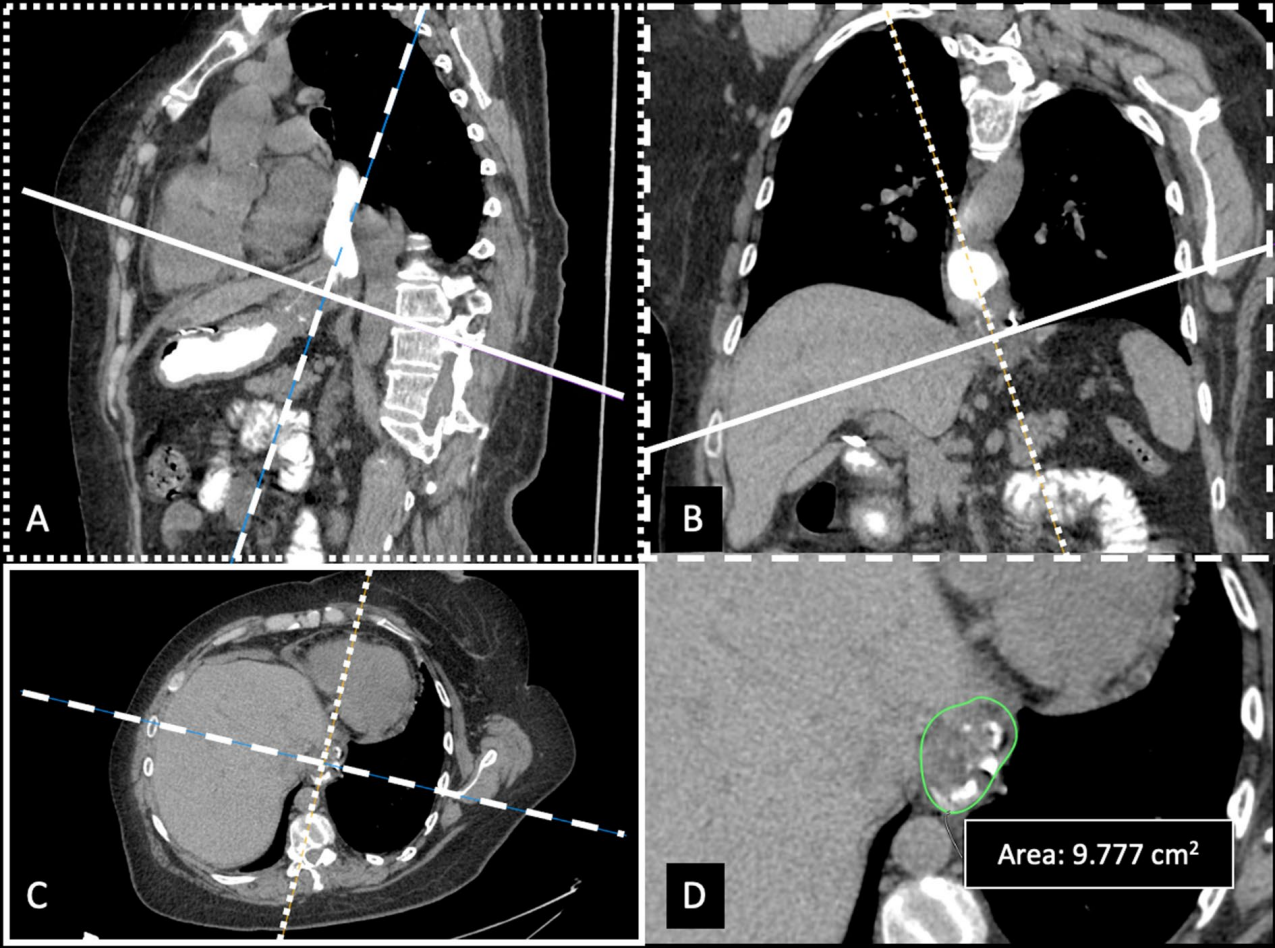
HSA measure on MPR reconstruction. The procedure begins from the three conventional MPRs (axial, sagittal and coronal) with cross-reference lines displayed. As a first step, the reference line of the axial image (solid line) is moved and rotated such that it intersects the anterior and posterior margins of the esophageal hiatus on the sagittal plane (A). Since MPRs are in a fixed orthogonal position, also the coronal (B) and axial planes are modified. Afterward, the solid line is rotated such that it intersects the right and left margins of the esophageal hiatus on the coronal plane (B). The resulting doubly oblique axial plane (C) is exactly parallel to the esophageal hiatus. Finally, the area of the esophageal hiatus is measured, drawing a polygonal hand-crafted ROI (region of interest) to define the inner margin of the hiatus using the fat–crural interface (D)

**Fig. 3:**
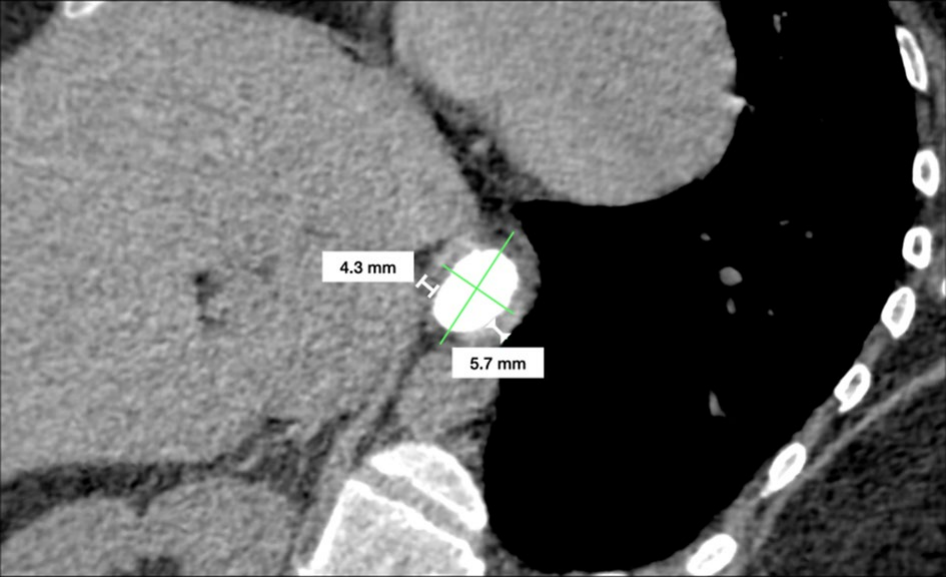
Diaphragmatic pillars (DP) measurement on MPR reconstruction. On this para-axial image, the entire hiatal area is represented. Diaphragmatic pillars are measured (withe lines) at the posterior third of the hiatus

**Fig. 4:**
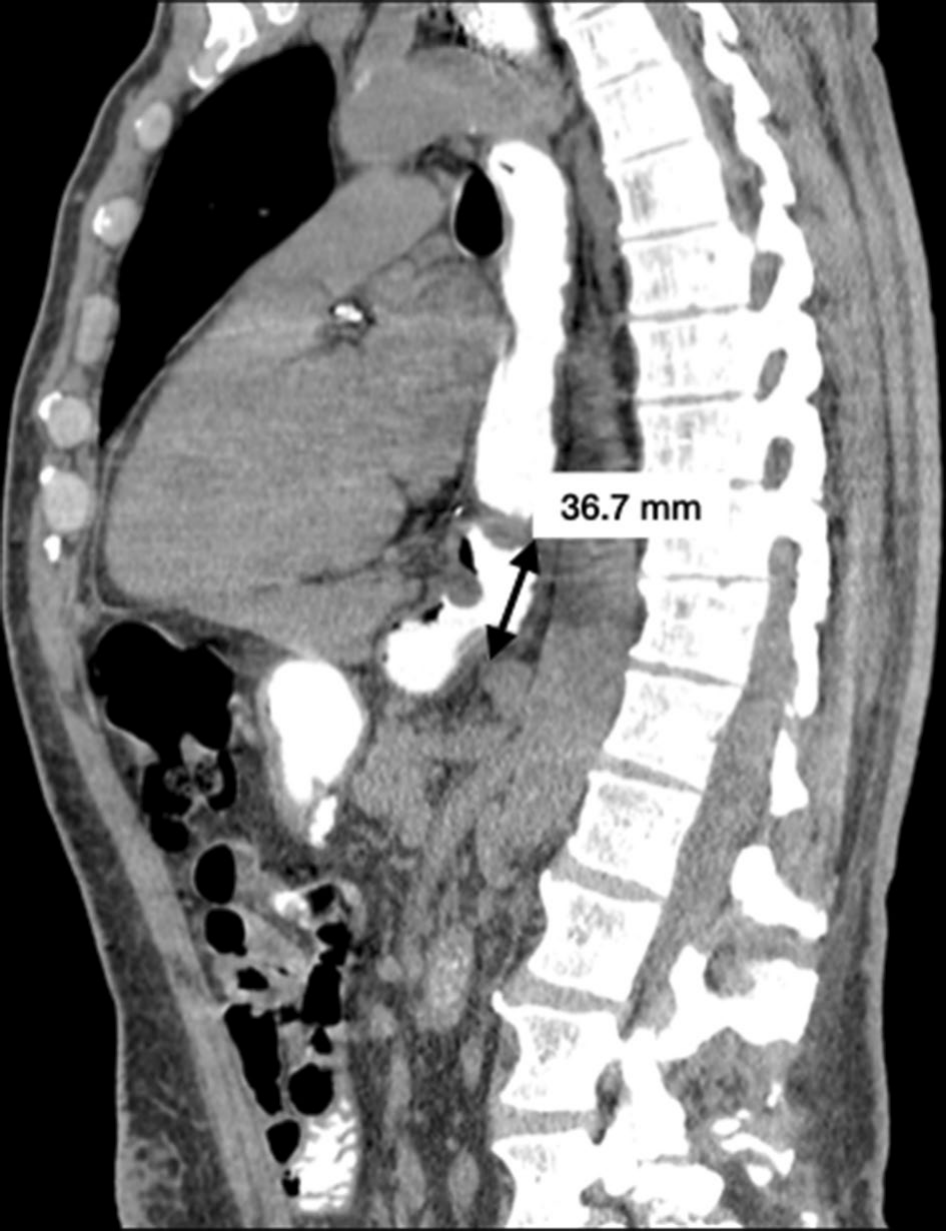
Intrathoracic Migration of gastroesophageal junction. On this para-sagittal image, the distance between the gastroesophageal junction and the diaphragmatic hiatus plane is measured (black line with solid arrows)

All datasets were anonymized and transferred on a dedicated workstation (Advantage Window 4.6, GE Medical Systems®, Waukesha, WI, USA). Multiplanar reconstructions (MPR) were obtained from raw data and used to obtain a double-oblique-corrected plane showing the esophageal hiatus as previously described [13, 20]. HSA was measured using a polygonal hand-crafted region of interest (ROI) (Fig. 2). On this dedicated plane, the maximum thickness of diaphragmatic pillars was measured (Fig. 3). Finally, as previously described [13], a sagittal plain was reconstructed to measure the migration of GEJ from the diaphragmatic hiatus plane (Fig. 4). All measurements were performed on both swallowing and straining acquisitions.

All datasets were analyzed by nine independent readers divided in three groups. Group 1 was composed of one expert radiologist with more than 10 years of experience in imaging of bariatric patients. Group 2 was composed of four radiologists with more than five years of experience in abdominal imaging and no specific training on bariatric patients. Group 3 was composed of four residents in radiology with one year of experience in abdominal imaging and without training on bariatric patients.

### Surgical procedures

According to symptoms, clinical evaluation and IWL patients were suggested to laparoscopic hiatal hernia repair (HHR) as a stand-alone procedure or combined with re-sleeve gastrectomy, Roux-en-Y gastric bypass conversion (RYGB) or one anastomosis gastric bypass conversion (OAGB/MGB).

#### HSA measurement

**Fig. 5:**
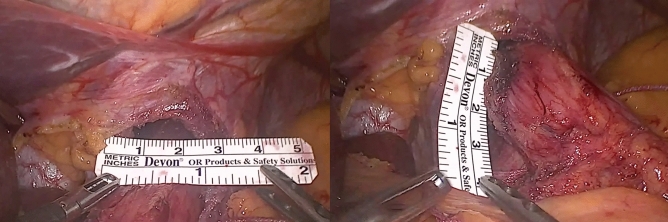
Intraoperative calculation of HSA. Intraoperative measurement of HSA by using simplified rhomb formula (R x S)/2 after complete pillar dissection and creation of retroesophageal window. R represents the length of the crura from the beginning at the crural commissure up to the superior edge of the esophageal hiatus. S represents the major horizontal distance between the two crura including their thickness

A ruler was routinely used intraabdominally for the intraoperatively HSA measurement, regardless the concomitant bariatric procedure: The length of the crura is measured in centimeters beginning at the crural commissure up to the superior edge of the esophageal hiatus (radius; R) after a completed HSA exposure and EGJ intraabdominal reduction. Then, the major horizontal distance between the two crura, including their thickness, is measured (S). With these two values, a simplified rhomboid formula was used as previously described [21, 22] (Fig. 5).

$$ HSA = \frac{{\left( {R \times S} \right)}}{2} $$

#### Posterior cruroplasty technique

Patients were divided in two subgroups, based on intraoperative HSA measurement:

- HSA ≤ 4 cm^2^: simple PC by 2–3 interrupted, nonabsorbable sutures, calibrated on a 42 French bougie, providing an efficient closure, with the esophagus lying loose through the hiatus.
- HSA > 4 cm^2^: reinforced PC with additional application of a biosynthetic, absorbable mesh containing a copolymer of polyglycolic acid and trimethylene carbonate (Bio-A®, Gore®, Flagstaff, USA). The mesh was fixed with absorbable stitches on each of the lateral sides on both right and left crus and with further application of glue (Evicel®, Johnson & Johnson) [24].

### Statistical analysis

First, a calculation of the optimal sample size was performed. Previous studies [13, 14] demonstrated that the HSA of patients without HH is inferior to 2.5 cm^2^, while the HSA of patients with HH is superior to 6.9 cm^2^. Moreover, if HSA is inferior to 4 cm^2^ a simple suture is sufficient to treat surgically HH, while if HSA is superior to 4 cm^2^ a reinforced suture is needed to treat HH [23]. To reach a power of 0.99 and a two-tailed α of 0.01, at least 19 subjects need to be included in the analysis.

Continuous variables were tested for normal distribution using the Kolmogorov–Smirnov test.

HSA measured on MDCT and intraoperatively, expressed in square centimeters, was compared using Friedman test with Dunn’s multiple comparison test. The correlation of the two measurements was also calculated using the Spearman correlation coefficient. Both tests were performed comparing surgical measurements with MDCT measures calculated on swallowing and straining acquisitions separately by each group. In groups 2 and 3, measures of all readers were averaged.

MDCT measurements (HSA, ITM and thickness of diaphragmatic pillars) reproducibility was assessed using inter- and intrareader agreement.

The interreader agreement was evaluated for group 2 and group separately 3 by means of intraclass correlation coefficient (ICC).

The intrareader agreement was evaluated for groups 1, 2 and 3 separately by means of weighted Cohen’s kappa (κ) analysis.

Agreement was interpreted according to the following criteria: > 0.81: excellent agreement; 0.61–0.80: good agreement; 0.41–0.60: moderate agreement; 0.21–0.40: fair agreement; and  < 0.20: poor agreement.

A correlation between HSA and diaphragmatic pillars thickness or GEJ’s motility was assessed using the Pearson correlation coefficient. For this analysis, MDCT measurements performed by group 1 were used. The diaphragmatic pillar thickness was estimated as the average of right and left pillars maximum thickness measured on MDCT during swallowing. The GEJ’s motility was expressed in percentage as the difference between the ITM, expressed in millimeters, measured on MDCT during swallowing and strain (Fig. 4) using the following equation:

$$ \frac{ITM \;during\; strain\; - ITM\; during\; swallowing}{{ITM\; during \; strain}} \times 100 $$

All continuous variables were expressed as mean ± standard deviation (SD), and a *p* value of < 0.05 was considered as statistically significant.

Statistical analyses were performed with GraphPad prism 5.0 (GraphPad Software®, La Jolla, CA, USA) and MedCalc (MedCalc Software® version 12.5, Ostend, Belgium).

## Results

### Study population

Seventy-two consecutive patients, candidates to revisional surgery after LSG between 2013 and 2020 for GERD, IWL and/or ITM, who underwent preoperative MDCT, were primarily included. Sixteen subjects were considered not eligible for this study due to (1) suboptimal imaging; (2) movement artifacts; (3) swallow problems; and (4) no HH. Four patients refused to participate to the study, so a final population of fifty-two patients was included in the analysis (Fig. 1).

The median age of participants in study was 47 years (age range 37–61), and 37 of them (72%) were women. The mean BMI at the primary LSG was 45.6 ± 7.5 kg/m^2^; the nadir BMI was 29.2 ± 4.8 kg/m^2^, while BMI at revision was 32.7 ± 6.4 kg/m^2^. Upper GI endoscopy demonstrated esophagitis LA A in 13, LA B in 6 cases and LA C in 4, while no case of Barrett’s esophagus was registered. One case of cardiac metaplasia without goblet cells was detected 4 years postoperatively.

### Surgical data

The median value of HSA, measured intraoperatively, was 5.34 cm^2^ (SD ± 2.82). HSA was smaller than 4cm^2^ (3.09 cm^2^ ± 0.55) in 27 patients (51.9%) and larger (7.77 cm^2^ ± 2.18) in 25 patients (48.1%). All surgical procedures were completed laparoscopically. A PC was performed as stand-alone procedure in 15 patients (28%); 5 simple PC (9.6%) and 10 reinforced PC (19.2%). The remaining 37 patients (72%) were subjected to laparoscopic hiatal hernia repair (PC simple or reinforced) concomitant with different revisional bariatric procedures: re-sleeves in 10 patients, RYGB conversion in 25 patients and OAGB/MGB conversion in 2 patients. Results are summarized in Table 1.

**Table 1:** Laparoscopic hiatal hernia repair procedures performed after sleeve gastrectomy

HHR (stand-alone procedure)	No of cases (%)
Simple PC (HSA < 4cm^2^)	5 (9.6)
Reinforced PC (HSA > 4 cm^2^)	10 (19.2)
*Bariatric procedures with concomitant HHR*	
Re-sleeve + simple PC	5 (9.6)
Re-sleeve + reinforced PC	5(9.6)
RYGB + simple PC	15 (28.9)
RYGB + reinforced PC	10 (19.2)
OAGB/MGB + simple PC	2 (3.9)
Total patients	52 (100)

Laparoscopic hiatal hernia repair (HHR). Posterior cruroplasty (PC). R-en-Y gastric bypass (RYGB). Single anastomosis gastric bypass (OAGB/MGB). Percentage of the total population in parenthesis

### HSA: MDCT Vs Intraoperative measurement

Results are summarized in Table 2.

**Table 2:** HSA measurement. Comparison between MDCT and intraoperative measurements

*HSA measurement*			
	HSA mean	HSA ≤ 4 cm^2^	HSA > 4 cm^2^
*Intraoperative*			
Patients	52	27	25
Area	5.34 ± 2.82	3.09 ± 0.55	7.77 ± 2.18
*MDCT*			
Group 1	52	27	25
Swallowing	5.23 ± 2.87	2.98 ± 0.53	7.66 ± 2.32
*p* value*	0.384	0.175	0.438
r**	0.9404	0.6767	0.8701
	(0.89–0.96)	(0.39–0.84)	(0.72–0.94)
Strain	5.68 ± 2.96	3.16 ± 0.42	8.41 ± 1.87
*p* value*	0.475	0.325	0.561
r**	0.9499	0.6749	0.9577
	(0.91–0.97)	(0.38–0.84)	(0.90–0.98)
Group 2	52	27	25
Swallowing	5.08 ± 2.62	3.03 ± 0.82	7.28 ± 2.13
*p* value*	0.258	0.037	0.028
r**	0.9303	0.659	0.8011
	(0.88–0.96)	(0.36–0.83)	(0.58–0.91)
Strain	5.68 ± 3.11	3.04 ± 0.59	8.53 ± 1.97
*p* value*	0.372	0.137	0.425
r**	0.9357	0.6285	0.8886
	(0.88–0.96)	(0.31–0.81)	(0.75–0.95)
Group 3	52	27	25
Swallowing	5.15 ± 2.53	3.15 ± 0.75	7.29 ± 2.02
*p* value*	0.147	0.025	0.019
r**	0.8909	0.3927	0.7807
	(0.81–0.94)	(0.15–0.67)	(0.55–0.90)
Strain	5.66 ± 3.07	3.06 ± 0.55	8.47 ± 1.98
*p* value*	0.125	0.078	0.247
r**	0.9235	0.6169	0.7931
	(0.86–0.95)	(0.29–0.81)	(0.57–0.90)

HSA (hiatal surface area) expressed in cm^2^ (± SD). *calculated using Friedman test with Dunn’s multiple comparison test. ** calculated using Spearman correlation coefficient. 95% confidence intervals in parenthesis

All readers correctly classified patients as HSA ≤ 4cm^2^ or > 4 cm^2^ as determined by the intraoperative measurement.

When HSA, measured on MDCT and intraoperatively, was compared, significant differences were observed only for measurements performed on swallowing imaging for groups 2 and 3 for small (≤ 4cm^2^; *p* = 0.037 and 0.025) and large (> 4 cm^2^; *p* = 0.028 and 0.019) areas. No other statistically significant differences were observed (*p* > 0.05).

All Spearman correlation coefficients were statistically significant (*p* < 0.05). An excellent correlation between HSA measured intraoperatively and on MDCT acquired during strain was observed in all groups (r ranging between 0.94 and 0.92), while an inferior correlation was observed comparing measurements performed on MDCT acquired during swallowing (r ranging between 0.94 and 0.89). This trend was observed also for HSA ≤ 4cm^2^ and > 4 cm^2^ in all groups.

All the correlations between intraoperative and MDCT HSA measurements were higher for more experienced readers compared to less experienced ones. The same results were observed comparing HSA ≤ 4cm^2^ and > 4 cm^2^ where correlations were higher when larger HAS was measured.

### Reproducibility

Results are summarized in Table 3. The overall reproducibility of MDCT HSA measurement, comparing all nine readers and both strain and swallowing acquisitions, was excellent resulting in a grouped ICC of 0.95 (95% CI 0,8993 to 0,9840) independently of reader’s experience.

**Table 3:** Reproducibility of measurements with intra- and interreader agreement

	HSA	ITM	DP Right	DP Left
Group 1** Intrareader	0.93	0.97	0.83	0.75
	(0.86–1.00)	(0.95–1.00)	(0.78–0.94)	(0.61–0.91)
Group 2* Interreader	0.93	0.96	0.79	0.98
	(0.84–0.97)	(0.91–0.98)	(0.53–0.93)	(0.96–1.00)
Group 2** Intrareader	0.82	0.84	0.88	0.96
	(0.59 –0.94)	(0.63 –0.96)	(0.69 –0.98)	(0.90–0.98)
Group 3* Interreader	0.89	0.94	0.54	0.52
	(0.75 -0.96)	(0.87 –0.98)	(0.39–0.83)	(0.35–0.78)
Group 3** Intrareader	0.83	0.98	0.64	0.67
	(0.60 –0.95)	(0.96—0.99)	(0.13–0.88)	(0.37–0.85)

*HSA* hiatal surface area, *ITM* intrathoracic migration, *DP* diaphragmatic pillar. 95% confidence intervals in parenthesis. * Estimated by intraclass correlation coefficient. ** Estimated by weighted Cohen’s kappa

The interreader agreement, for HSA measurement, among group 2 (ICC of 0.93; 95% CI: 0,8461 to 0,9777) was higher than among group 3 (ICC of 0.89; 95% CI 0.7574 to 0.9666).

An excellent intrareader agreement for HSA measurement was also found in all groups (κ = 0.93, 0.82 and 0.83).

The reproducibility of the other measurements (ITM and diaphragmatic pillars thickness) was high. A good-to-excellent intra- and interreader agreement was found for all measurement. Reader’s experience level influenced the results since experienced readers obtained a higher agreement for all measurements.

### Diaphragmatic pillars and gastroesophageal junction migration

Right and left diaphragmatic pillars were measured on the dedicated MDCT plane used to measure HSA. In this study, mean values were 6.64 ± 1.57 mm for right crus and 7.01 ± 1.27 mm for the left one. The mean diaphragmatic pillars thickness was 6.03 ± 1.93 mm. A very low correlation between HSA and the mean diaphragmatic pillars thickness was observed (r = 0.1365; 95% CI − 0.215 to 0.772; *p* = 0.2372).

ITM of GEJ was demonstrated in all patients on the dedicated sagittal MPR. An average migration of 36.12 ± 16.93 mm (distance between the GEJ and the diaphragmatic hiatus) was observed in this population. The mean ITM was 34.57 ± 20.11 mm during swallowing and 39.29 ± 25.19 mm during strain acquisition. The average GEJ’s motility was 13.67% ± 3.12. A very low correlation between HSA and GEJ’s motility was observed (r = 0.05912; 95% CI: -3.591 to 1.706; p = 0.4464).

## Discussion

The present study demonstrated an excellent agreement between HSA measured intraoperatively and on MDCT images. To the best of our knowledge, this is the first radiological study conducted on morbid obese patients subjected to LSG, demonstrating the correlation between preoperative noninvasive measurement simulating surgical condition and intraoperative measurement of HSA. However, the method used is not completely original since, for the HSA measurement on MDCT images, we used the same technique described in two previous studies [13, 20]. The main difference between our method and previous ones is the use of a dedicated acquisition technique which requires the administration of oral iodinated contrast media and image acquisition during swallowing and strain. Moreover, one of the two studies [13] defined the correlation between HSA, measured on MDCT images and the presence of HH, using a standard CT protocol (no oral contrast nor dynamic acquisitions) and without the intraoperative measurements as reference standard. The study of Moten AS et al. [20], instead, compared the measurement of HSA on MDCT and intraoperatively using a standard CT protocol in nonobese patients candidate to HHR. The results were comparable to ours but a lower correlation between the two methods was observed (r = 0.83 Vs r = 0.94/0.89), suggesting the added value of dynamic acquisitions, especially the scan during strain.

In the present study, we found a difference between measurements performed on swallowing and strain images. A higher correlation between intraoperative and MDCT HSA measures was observed for strain images in all groups. Moreover, for less experienced readers (group 2 and 3) a significant difference was observed when measuring HSA ≤ 4cm^2^ or > 4 cm^2^ compared to intraoperative results. This was particularly evident, when measuring HSA ≤ 4cm^2^, since a lower correlation (r = 0.65 and 0.39) was also observed. The better results obtained with strain imaging may be explained since, during strain, it is simulated the effect of the positive intraabdominal pressure induced by pneumoperitoneum used in laparoscopic procedure. We also believe that the use of oral contrast media and strain imaging may give additional information on the anatomy of such a complex structure like gastroesophageal junction and esophageal hiatus. To confirm that, a direct comparison between our method and a simple measurement performed on any MDCT study should be performed.

We also investigated whether our method may give additional information to the surgeon for surgical planning optimization. The status of the diaphragmatic crura, in particular the ultrastructure alteration of the diaphragmatic pillars, was supposed to influence the outcome of the cruroplasty [24]. However, we were not able to find a correlation between HSA and diaphragmatic pillars thickness. Probably the ultrastructure alteration of such structures cannot be established on the basis of their thickness only. Moreover, we didn’t find a correlation between HSA and gastroesophageal junction mobility measured with MDCT.

Since the imaging procedure proposed in the present study was not validated yet, its accuracy may be influenced by the experience of the radiologist performing the measurement. For this reason, we stratified our results according to readers’ experience. We observed that both experienced and less experienced readers obtained an excellent correlation with intraoperative HSA measurement. More importantly, HSA was correctly classified according to intraoperative classification independently from reader’s experience.

We also confirmed that this method is highly reproducible since good-to-excellent intra- and interreader agreement was observed [13]. Furthermore, we observed that reproducibility has been minimally influenced by reader experience since both experienced and less experienced readers obtained a good agreement.

Our study has some limitations. First, we didn’t investigate the effect of surgery on symptoms or the relapse of hiatal hernia. This would be important to understand whether MDCT can influence the surgical technique. We used a simplified formula to calculate HSA intraoperatively, validated in previous series [22], demonstrating a difference < 10% compared with the more complex Granderath’s formula [23] that does not change the HSA’s classification. Another limit of this study is the single-center design since a multicentric study should be advisable. However, we can consider this as a pilot study. Finally, we investigated the accuracy of this method only on patients previously subjected to LSG; however, we believe that similar results can be obtained also on naïve patients or on patients subjected to other bariatric procedures.

In conclusion, we demonstrated that, using the proposed method, HSA can be accurately measured on MDCT images and that this approach is reproducible and minimally influenced by reader’s experience. The use of strain acquisition increases the accuracy of the HSA measurement and thus should be routinely acquired for such evaluation. An accurate preoperative measurement of HSA may have several advantages for surgeons and patients. First, the surgical technique may be decided preoperatively. Surgeons may choose to perform a simple PC in case of HSA ≤ 4 cm^2^ or a reinforced PC, with biosynthetic mesh, in case of a greater areas. The surgical technique can be more accurately discussed with the patient in advanced. Second, the time consumed for the intraoperative calculation of the HSA can be saved. Laparoscopic procedures have the advantages, compared to laparotomic ones, of a shorter operative time which can be further reduced avoiding the time-consuming dissection of the hiatal area required to measure the HSA. Thus, we recommend noninvasive HSA measurement by MDCT before laparoscopic revisional surgery after LSG in case of severe GERD due to hiatal hernia and intrathoracic migration.

## Abbreviations

LSG: Laparoscopic sleeve gastrectomy
GERD: Gastroesophageal reflux disease
ITM: Intrathoracic migration
HH: Hiatal hernia
LES: Low esophageal sphincter
HSA: Hiatal surface area
MDCT: Multidetector computed tomography
PPI: Proton pump inhibitors
IWL: Insufficient weight loss
GERD-HRQL: GERD-Health Related Quality of Life
EGD: Esophagogastroduodenoscopy
GEJ: Gastroesophageal junction
MPR: Multiplanar reconstructions
HHR: Laparoscopic hiatal hernia repair
RYGB: Roux-en-Y gastric bypass
OAGB/MGB: One anastomosis gastric bypass/mini-gastric bypass
BMI: Body mass index
ROI: Region of interest
DP: Diaphragmatic pillars
SW: Swallow
ST: Strain
ICC: Intraclass correlation coefficient
